# The Adenomatous polyposis coli tumour suppressor is essential for Axin complex assembly and function and opposes Axin's interaction with Dishevelled

**DOI:** 10.1098/rsob.110013

**Published:** 2011-11

**Authors:** Carolina Mendoza-Topaz, Juliusz Mieszczanek, Mariann Bienz

**Affiliations:** MRC Laboratory of Molecular Biology, Hills Road, Cambridge CB2 0QH, UK

**Keywords:** APC tumour suppressor, Axin degradasome, Dishevelled signalosome

## Abstract

Most cases of colorectal cancer are linked to mutational inactivation of the Adenomatous polyposis coli (APC) tumour suppressor. APC downregulates Wnt signalling by enabling Axin to promote the degradation of the Wnt signalling effector β-catenin (Armadillo in flies). This depends on Axin's DIX domain whose polymerization allows it to form dynamic protein assemblies (‘degradasomes’). Axin is inactivated upon Wnt signalling, by heteropolymerization with the DIX domain of Dishevelled, which recruits it into membrane-associated ‘signalosomes’. How APC promotes Axin's function is unclear, especially as it has been reported that APC's function can be bypassed by overexpression of Axin. Examining *apc* null mutant *Drosophila* tissues, we discovered that APC is required for Axin degradasome assembly, itself essential for Armadillo downregulation. Degradasome assembly is also attenuated in *APC* mutant cancer cells. Notably, Axin becomes prone to Dishevelled-dependent plasma membrane recruitment in the absence of APC, indicating a crucial role of APC in opposing the interaction of Axin with Dishevelled. Indeed, co-expression experiments reveal that APC displaces Dishevelled from Axin assemblies, promoting degradasome over signalosome formation in the absence of Wnts. APC thus empowers Axin to function in two ways—by enabling its DIX-dependent self-assembly, and by opposing its DIX-dependent copolymerization with Dishevelled and consequent inactivation.

## 2. Introduction

Individuals carrying a germline mutation in the Adenomatous polyposis coli (APC) tumour suppressor almost invariably develop colorectal cancer by mid-life, and the majority of sporadic colorectal cancers are also linked to *APC* mutations [1]. APC is a highly conserved negative regulator of Wnt/β-catenin signalling that cooperates with Axin to destabilize the key effector of this pathway, β-catenin (Armadillo in *Drosophila*) [2–4]. Loss of APC thus causes stabilization of β-catenin, which results in its hyperactivation. If this occurs inappropriately in the intestinal epithelium, tumourigenesis is initiated, apparently because the hyperactive β-catenin in the *APC* mutant cells confers stem cell-like properties on them [5]. APC also controls many cell fate decisions in normal animal development by keeping the levels of its β-catenin/Armadillo effector low [6].

The molecular role of Axin as a constitutive negative regulator of Wnt signalling is well understood: Axin is a scaffold protein that binds directly to β-catenin/Armadillo and to glycogen synthase kinase 3 (GSK3), to enable this kinase to phosphorylate its substrate [7,8], by increasing the efficiency of this enzymatic reaction by several orders of magnitude [9], consequently targeting β-catenin/Armadillo for proteasomal degradation. This function of Axin depends crucially on its C-terminally located DIX domain (hereafter referred to as DAX) [10–12], which mediates rapid and reversible polymerization [13], and thus allows assembly of a dynamic interaction platform with high avidity for low-affinity binding partners [14]. These large DAX-dependent multi-protein complexes are visible in cells as prominent puncta, and they reflect functional Axin destruction complexes (hereafter referred to as ‘degradasomes’): they contain other components of this complex such as APC and GSK3, and they promote the destabilization of β-catenin/Armadillo [10,11].

The constitutive function of Axin in destabilizing β-catenin/Armadillo is blocked upon Wnt signalling [15,16], which triggers Axin's relocation to the plasma membrane (PM) by Dishevelled (Dsh in flies, Dvl in mammals) [17,18]. This interaction depends on Dishevelled's own DIX domain (hereafter referred to as DIX), which binds directly to Axin DAX [11]: indeed, heterotypic DIX–DAX interactions involve the same DAX surface residues as those mediating homotypic DAX–DAX polymerization, and so the two interactions are mutually exclusive. Thus, the heterotypic copolymerization with Dishevelled is in direct competition with the DAX-dependent homopolymerization of Axin, which in turn is essential for Axin's degradasome function [11]. In other words, Axin DAX is pivotal for switching β-catenin signalling from OFF (requiring DAX–DAX homopolymerization) to ON (mediated by DAX–DIX heteropolymerization with Dishevelled).

Axin has one other structural domain in its N-terminus (called RGS domain), through which it binds directly to APC [19–23], but how this interaction contributes to Axin's effector function is unclear. Indeed, despite its abovementioned crucial role as a tumour suppressor, APC can be dispensable for the downregulation of β-catenin/Armadillo under certain conditions, most notably in colorectal cancer cells that express APC truncations (retaining partial *APC* function; see below): if Axin or Axin2/Conductin are overexpressed in these *APC* mutant cancer cells, they are capable of destabilizing β-catenin, thereby restoring low Wnt pathway activity [19,20,22]. Indeed, a recent study (predominantly involving Axin overexpression) concluded that APC is not absolutely required for the formation of a functional destruction complex [10]. Likewise, overexpression of *Drosophila* Axin reverses the ‘naked’ embryonic phenotype caused by an *apc* point-mutation (called *N175K*) towards normal [18]. High levels of overexpressed Axin can thus compensate for dysfunctional APC in flies and human cells, suggesting that APC acts through Axin by increasing its efficiency in destabilizing β-catenin/Armadillo rather than executing an integral core function in this process. Notably, Axin is thought to be rate-limiting as its cellular levels are low when compared with those of other Wnt signalling components [24], so Axin may depend on APC for functional efficiency primarily because of its low abundance.

Here, we revisit the regulatory relationship between APC and Axin, noting that all previous tests of Axin function were done under partial *APC* loss-of-function conditions: virtually all *APC* mutations found in cancers produce APC truncations whose Axin-binding sites are deleted, but which retain multiple β-catenin-binding modules [25], which could confer partial Wnt pathway activity, conducive to tumourigenesis [26]. Indeed, typical APC cancer truncations even retain the ability to associate with Axin [20], possibly through other factors (see also [27,28]). Likewise, in *Drosophila*, previous tests of Axin function [18] were done with an *apc* point-mutant allele that causes predominantly a mislocalization of APC [29]. However, *Drosophila apc* null mutants have since been isolated and characterized [30], which prompted us to re-examine the activity of Axin in a true *apc* null mutant background. We thus discovered that APC is indispensable for the assembly of functional Axin degradasomes. The same also appears to be true in *APC* mutant colorectal cancer cells. Furthermore, we present evidence based on *apc* null mutant *Drosophila* tissues and on co-overexpression assays in human cells that APC functions to oppose the interaction of Axin with Dishevelled, thus shielding it from Dishevelled-mediated inactivation. This suggests that APC enables Axin to function in two ways—by imposing Axin homopolymerization, and by opposing its heteropolymerization with Dishevelled.

## 3. Results

### 3.1. Adenomatous polyposis coli is essential for Axin degradasome assembly and function in *Drosophila* embryos

*Drosophila* contains two *APC* genes, one expressed ubiquitously (called *E-APC*/*Apc2* [31,32]), for which a null allele became available recently (*e-apc*^*f*^^90^ [30]), and another one predominantly expressed in neurons, for which a null allele was isolated and characterized many years ago (*dApc*^*Q*^^8^ [33]). To re-assess the role of APC in promoting Armadillo destabilization, we monitored the expression and function of Axin tagged with green fluorescent protein (Axin–GFP) in embryos completely lacking the function of both *APC* genes (*apc* null mutants; *dApc*^*Q*^^8^*e-apc*^*f*^^90^) using the GAL4 system as previously described [18].

**Figure 1.**
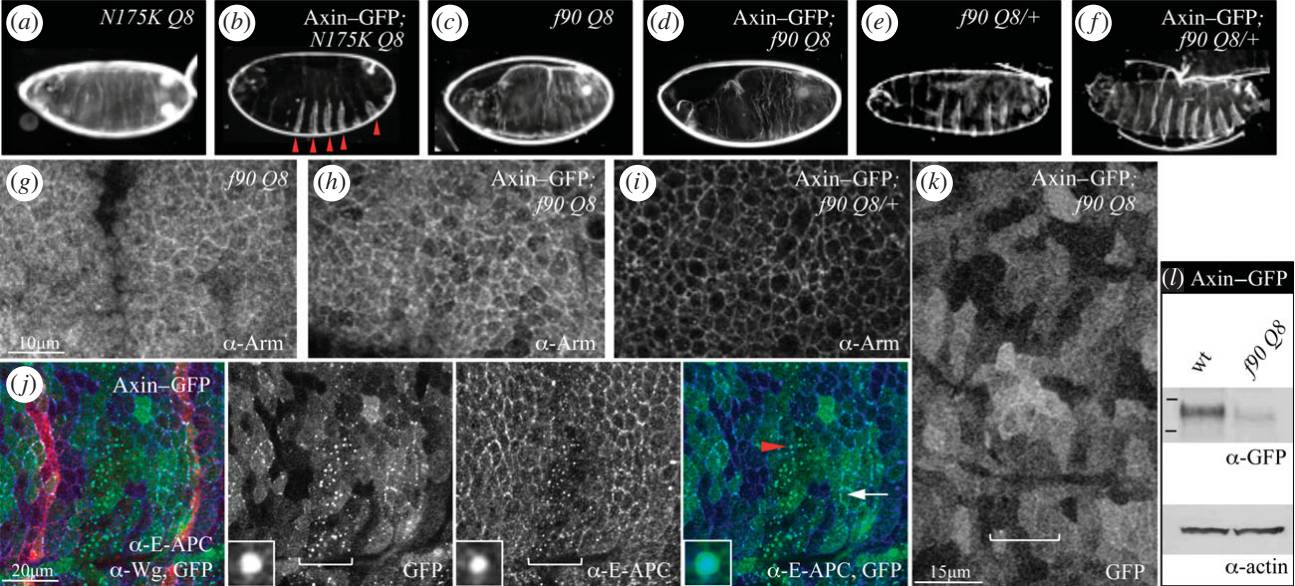
APC is essential for the assembly of functional Axin–GFP degradasomes in *Drosophila* embryos. (*a*–*f*) Ventral embryonic cuticles derived from *apc* germline clones (*N175K Q8, e-apc hypomorph; f90 Q8, apc* null) expressing Axin–GFP (with *arm.GAL4*), (*a*–*d*) without or (*e*,*f*) with zygotic APC; (*b,d,f*) arrowheads indicate denticle belts, signifying degradasome function of Axin–GFP (see figure 4*a* for normal cuticle); every single Axin–GFP-positive *apc* null mutant embryo is naked (*d*). (*g*–*i*) Single confocal sections through the apical lateral epidermis (parallel to the epithelial plane) of 4–5-h-old embryos (genotypes as in *c,d,f*), fixed and stained with α-Armadillo; (*h*) *apc* null mutants show high levels of cytoplasmic Armadillo, regardless of Axin–GFP, signifying lack of Axin–GFP function, while (*i*) sibling controls show low levels of cytoplasmic Armadillo (but high-junctional Armadillo, which is not downregulated by Axin–GFP). (*j*) Single confocal section as in (*g*–*i*) of an approximately 6-h-old wt embryo expressing Axin–GFP (green), co-stained for E-APC (blue) and Wg (red); single channels in the middle, double- and triple-merges on the outside; arrow points to small Dsh-dependent Axin–GFP puncta at the PM (signalosomes), arrowhead to larger cytoplasmic puncta (degradasomes) into which E-APC is recruited (as shown in insets at high magnification; punctum diameter, approx. 0.5 µM); zones of low Wg (where degradasomes form) between Wg stripes (red in triple-merge) are bracketed. Note that the uneven GFP expression reflects the patchiness of the *arm.GAL4* driver [11,18]. (*k*) GFP channel from confocal section stained as in (*j*), but from *apc* null mutant embryo, revealing predominantly diffuse Axin–GFP (bracket as in *j*). (*l*) Western blot of total embryonic extracts, probed as indicated below panels, to reveal Axin–GFP levels in wt and *apc* null mutant embryos (bars, molecular weight markers for 188 and 98 kD).

We confirmed that Axin–GFP rescues the naked phenotype of hypomorphic *apc* mutants (*dApc*^*Q*^^8^*e-apc*^*N175K*^ [29]) by restoring normal-looking denticle belts [18] (figure 1*a,b*). Interestingly, though, Axin–GFP does not show any activity whatsoever in *apc* null mutants (figure 1*c*,*d*). Its activity in restoring normal denticle belts is manifest only in paternally rescued sibling controls (figure 1*e*,*f*), which express zygotic APC. The loss of Axin–GFP function in *apc* null mutants is 100 per cent penetrant: every single *apc* null mutant exhibits a completely naked cuticle (*n* = 102), without any sign of denticle restoration, demonstrating that APC is indispensable for Axin function in *Drosophila* embryos. Consistent with this, Axin–GFP does not reduce the high Armadillo levels in *apc* null mutant embryos (figure 1*g*,*h*), but only in paternally rescued sibling controls (figure 1*i*).

We previously found that Axin–GFP forms two sets of conspicuous puncta in the embryonic epidermis [11,18]: large cytoplasmic puncta, which contain apparently stoichiometric amounts of E-APC (expressed at high levels in epithelia [32]) and are thus likely to reflect Axin degradasomes (figure 1*j*, arrowhead and insets), and smaller Dsh-dependent PM-associated puncta in cells within the Wingless (Wg) signalling zones (likely to reflect signalosomes [17]) (figure 1*j*, arrow). Both sets of puncta depend on Axin's DAX domain [11]. Strikingly, Axin–GFP is largely diffuse in *apc* null mutant embryos (figure 1*k*), and is thus unable to assemble degradasomes in the absence of APC. We also find that the level of Axin–GFP is significantly reduced in these *apc* mutant embryos (to 35–38% of their level in wild-type (wt) embryos; figure 1*l*), which could contribute to the failure of degradasome assembly (see §4).

### 3.2. Axin degradasome assembly is inefficient in *apc* mutant colorectal cancer cells

**Figure 2.**
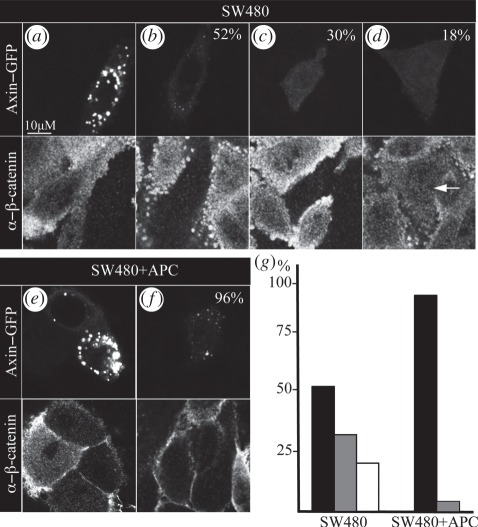
Failure of Axin–GFP degradasome assembly in *APC* mutant colorectal cancer cells. Single confocal sections through representative (*a*–*d*) *APC* mutant SW480 or (*e,f*) SW480 + APC rescue cells (see text) expressing Axin–GFP (top panels), fixed and stained with α–β-catenin (bottom panels), as indicated; (*a,e*) high-expressing cells, invariably showing punctate Axin–GFP; (*b*–*d*,*f*) low-expressing cells, showing (*b*) residual Axin–GFP puncta or (*c*,*d*) diffuse Axin–GFP, (*d*) often failing to downregulate β-catenin; (*f*) almost all low-expressing SW480 + APC rescue cells exhibit punctate Axin–GFP. (*g*) Graphical representation of percentages (also given in the corresponding panels, *b*–*d*,*f*) of low-expressing cells with residual Axin–GFP puncta (black bars), or diffuse Axin–GFP (grey bars, low β-catenin levels; white bars, high β-catenin levels).

In light of these results, we decided to revisit previous tests of Axin function in *APC* mutant cancer cells (typically done in SW480 cells [19,20,22]). Like most other *APC* mutant colorectal cancer cell lines, SW480 cells express an APC truncation that lacks its Axin-binding sites, although it retains the ability to associate with Axin, possibly indirectly [20]. As a control, we used the SW480 + APC rescue line bearing a stable wt *APC* transgene that restores the destabilization of β-catenin [34]. Both lines were transfected with low doses of Axin–GFP plasmid, to keep the overexpression minimal, which is optimal for functional read-outs [11]. If cells expressing high levels of GFP fluorescence are examined, these show punctate Axin–GFP, which is invariably accompanied by downregulation of β-catenin (figure 2*a*,*e*), as previously described [10,11] (note that the activity of Axin–GFP can only be monitored in the parental SW480 line, but not in the rescue line whose cytoplasmic β-catenin is destabilized owing to the *APC* transgene [34]).

However, if SW480 cells with the lowest detectable GFP fluorescence levels are selected for examination (*n* = 60; see §5.3), these exhibit largely diffuse Axin–GFP (figure 2*b*–*d*): although we can still detect minuscule Axin–GFP puncta in many of these (52%; *n* = 31), invariably accompanied by β-catenin downregulation (figure 2*b*), about half of these low-expressing cells (48%; *n* = 29) exhibit no discernable Axin–GFP puncta (figure 2*c*,*d*), and nearly one-third of the latter (*n* = 11) show no downregulation of β-catenin whatsoever (figure 2*d*). By contrast, if we select low-expressing SW480 + APC cells in the same way (*n* = 54), almost all of these (*n* = 50) show clearly visible Axin–GFP puncta (figure 2*f*), and only a few cells (*n* = 4) seem to be without puncta. These results (graphically represented in figure 2*g*) reveal a significant failure rate of Axin–GFP in assembling functional degradasomes in the *APC* mutant SW480 cells, although Axin–GFP is highly competent in assembling degradasomes in their matched control cells (regardless of expression levels). Given that the genetic background of these two cell lines is identical apart from their *APC* status, it indicates that the dysfunctional APC in the parental SW480 cells is responsible for the attenuated function of Axin–GFP. These results match those obtained in *apc* null mutant fly embryos, and suggest that APC is also required in human cells for efficient assembly of functional Axin degradasomes.

### 3.3. An Adenomatous polyposis coli-binding mutant of Axin fails to form functional degradasomes in *Drosophila*

To obtain independent evidence for the crucial role of APC in enabling Axin degradasome assembly in flies, we point-mutated two invariant residues in the APC-interacting surface of the *Drosophila* RGS domain (F87A G91E, called FGmut). The equivalent double-mutation in mammalian Axin abrogates its binding to APC *in vitro* [23]. RGS also exhibits a second conserved surface, corresponding to the G protein-interacting surface in *bona fide* RGS domains [23], and so we designed a second mutant in this alternative surface (E75K, D122E, D156E, N157A, EDDNmut), based on the structure of a known G protein interface [35], for comparison with FGmut. Two representative transgenic lines were selected for analysis on the basis of their expression levels comparable with that of our standard Axin–GFP line (see below).

**Figure 3.**
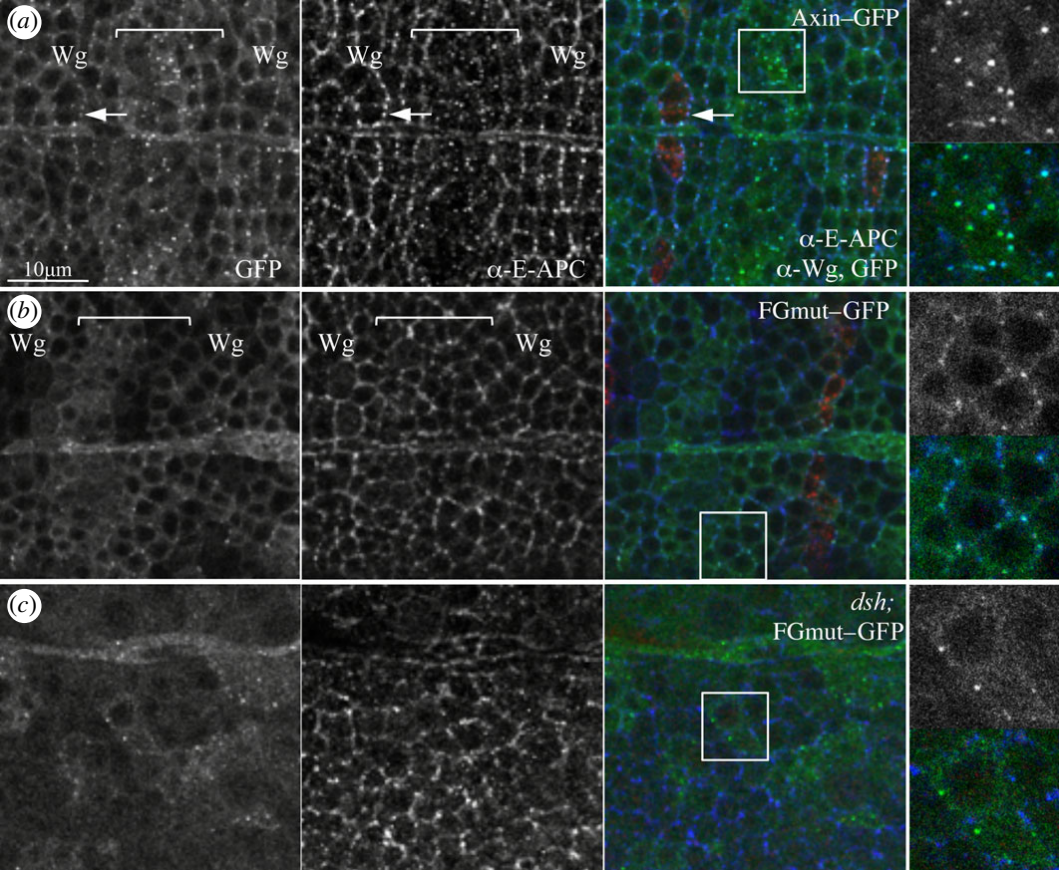
APC binding is required for efficient Axin–GFP degradasome assembly in *Drosophila* embryos. Single confocal sections through the embryonic epidermis as in figure 1*j*; (*a*,*b*) wt, (*c*) *dsh*^*v*^^26^ null mutant, expressing Axin–GFP or FGmut–GFP (green) as indicated, fixed and stained with α-E-APC (blue) and α-Wg (red); merges on the right. Arrows indicate signalosomes, bracketed are zones between Wg stripes with degradasomes (boxed areas are shown on the right, at high magnification, GFP above merge). Note the PM association of the residual FGmut puncta (*b*), and their cytoplasmic location in the *dsh* mutant (*c*).

EDDNmut exhibits a normal punctate expression pattern, indistinguishable from wt Axin–GFP (figure 3*a*), suggesting that this mutant is fully functional. In striking contrast, FGmut is largely diffuse (figure 3*b*) and forms far fewer puncta (some of which also stain for E-APC, suggesting that the binding of FGmut to E-APC is not completely blocked *in vivo*, or that FGmut resides in these puncta with E-APC by interaction with other factors; see also [27,28]). Notably, most of these FGmut puncta are relatively small and line the PM, even between Wg stripes (figure 3*b*), indicating that FGmut is prone to recruitment to the apical PM. We wondered whether this was owing to Dsh, given that the PM recruitment of the wt Axin–GFP puncta near the Wg stripes depends on *dsh* [18]. Indeed, FGmut appears even more diffuse in *dsh* null mutant embryos, and we only observed occasional puncta in the cytoplasm (figure 3*c*). This suggests that FGmut is prone to interact with Dsh, which could account for its residual activity to form PM-associated puncta. Its inability to form large cytoplasmic puncta is fully consistent with our result that wt Axin–GFP fails to assemble functional degradasomes in the absence of APC (figure 1*k*).

**Figure 4.**
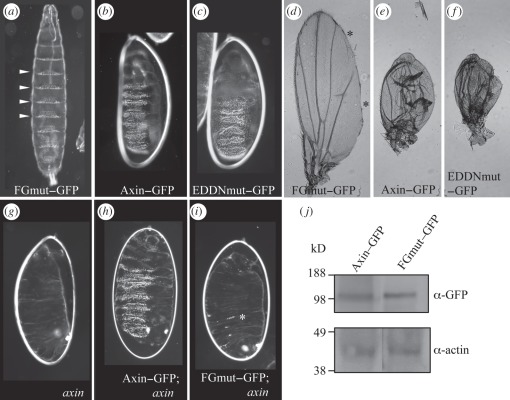
Loss of APC binding causes loss of Axin–GFP function in *Drosophila* tissues. (*a*–*f*) Overexpression of wt or mutant Axin–GFP in wt embryos and wing discs (as in figures 1 and 5, respectively), as indicated; (*a*–*c*) ventral larval cuticles (arrowheads point to normal denticle belts); (*d*–*f*) wings. FGmut is inactive, allowing (*a*) hatching of normal first instar larvae and (*d*) development of near-normal wings with only minor margin defects (asterisks), probably reflecting residual activity of this mutant. EDDNmut is as active as wt Axin–GFP, producing strong overexpression phenotypes as a result of downregulation of Armadillo in Wg signalling zones (see also text). (*g*–*i*) Rescue assays with Axin–GFP or FGmut–GFP after overexpression in *axin* null mutant embryos (as in figure 1; see also text); (*g*) *axin* null mutant embryo with completely naked cuticle; (*h*) Axin–GFP restores broad denticle belts, while (*i*) FGmut is almost completely inactive, restoring only occasionally individual denticles (example marked by asterisk). (*j*) Western blot of total embryonic extracts, probed as indicated next to panels, showing comparable levels of Axin–GFP and FGmut; internal control, actin (molecular weight markers on the left).

Notably, FGmut also lacks function as judged by overexpression assays: FGmut-overexpressing embryos are fully viable and hatch into aphenotypic larvae, and FGmut overexpression in wing discs produces only minor margin defects in wings (figure 4*a*,*d*). In contrast, EDDNmut causes a ‘denticle lawn’ phenotype upon overexpression in the embryo, followed by embryonic lethality, like wt Axin–GFP [18] (figure 4*b*,*c*), and severely attenuates wing blade development upon wing disc expression, leading to rudimentary wing stumps (figure 4*e*,*f*). The lack of FGmut function is further demonstrated in rescue assays of *axin* mutant embryos: these show naked cuticles (figure 4*g*) similar to *apc* mutant embryos (figure 1*c*), but overexpression of Axin–GFP restores denticle belts in each mutant embryo (*n* = 58; figure 4*h*), attesting a high level of rescue activity (given that the denticle fate depends on destabilization of Armadillo), as previously shown for this and other developmental contexts [11]. However, every single *axin* mutant embryo overexpressing FGmut (*n* = 57) is essentially naked, exhibiting only occasionally an individual denticle (figure 4*i*), thus reaffirming the inactivity of this mutant (whose expression level is comparable with that of Axin–GFP, as already mentioned; figure 4*j*). These overexpression and rescue assays demonstrate that Axin relies on direct binding to APC through its RGS domain for its function in antagonizing Armadillo signalling.

### 3.4. Absence of Adenomatous polyposis coli results in Dishevelled-dependent plasma membrane association and inactivity of Axin

Recall that Axin defective for binding to APC becomes prone to Dsh-dependent recruitment to the PM (figure 3*b*). Similar to this, we noticed that Axin–GFP shows a conspicuous lining of the PM in late-stage *apc* null mutant embryos (electronic supplementary material, figure S1*a*), in contrast to the hypomorphic *apc* mutants and to the wt (electronic supplementary material, figure S1*b*,*c*), but we were unable to test whether the PM-associated Axin–GFP in the *apc* null mutants depended on *dsh* as it was technically not feasible to generate double-null mutant embryos.

**Figure 5.**
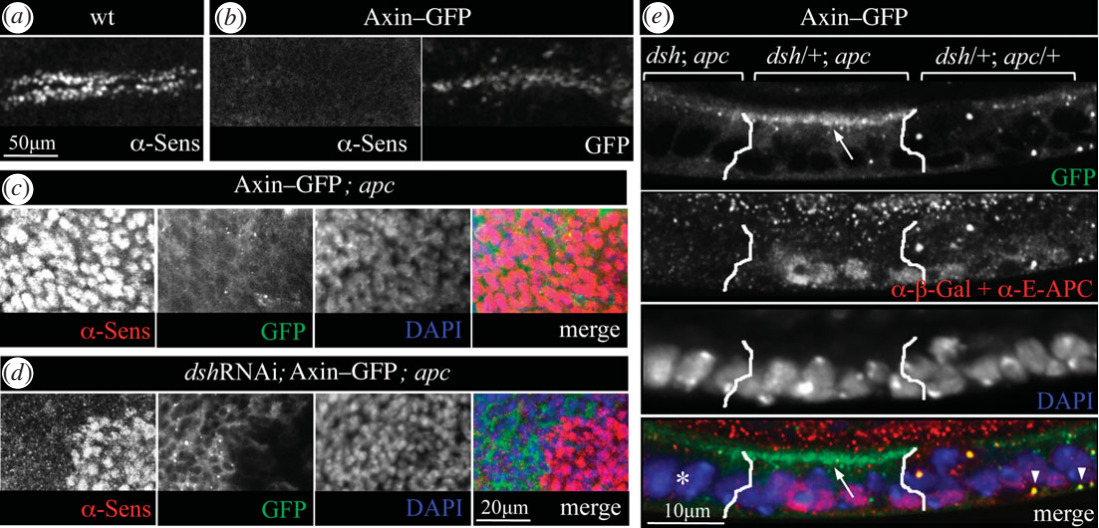
APC blocks Dishevelled-dependent PM relocation of Axin–GFP in wing imaginal discs. (*a*–*d*) Single confocal sections through the prospective margin area of wing discs from late third instar *Drosophila* larvae (parallel to epithelial plane), fixed and stained with antibodies as indicated; (*a*) wt disc, showing expression of the Wg target gene *sens* (flanking Wg expression along the margin); (*b*) *sens* repression by Axin–GFP (overexpressed with *vg.GAL4*); (*c*,*d*) high-magnification views of sub-apical sections through nuclei (marked by DAPI, blue) to reveal Sens expression (nuclear, red), (*c*) expressing Axin–GFP or (*d*) co-expressing Axin–GFP and *dsh*RNAi (green) with *vg.GAL4*; shown are boxed areas from discs shown in electronic supplementary material, figure S2*b*,*c*, selected for *apc* null mutant territories near the margin. Axin–GFP fails to repress *sens* in the absence of APC (*c*), but recovers repressive activity upon Dsh depletion (*d*, left in panel). Note that the GFP levels appear higher in (*d*) when compared with (*c*), due to Dsh-dependent recruitment of Axin–GFP to the apical PM (plane above these sections). (*e*) Single confocal section (perpendicular to epithelial plane) through a wing disc expressing Axin–GFP (near the Wg signalling zone, as in *c*); *apc* mutant cells (middle) are flanked by double-mutant (*dsh*; *apc*; left) and wt cells (right); note the large cytoplasmic degradasomes positive for both Axin–GFP and E-APC in the wt (marked by arrowheads in merge; see also electronic supplementary material, figure S2*a*) and the small apical Axin–GFP puncta (arrow), recruited to the PM by Dsh in the absence of APC; Axin–GFP puncta are rarely detectable in double-mutant cells (asterisk in merge).

We thus turned to wing discs in which we were able to deplete Dsh by overexpressing *dsh*-specific RNAi (*dsh*RNAi). Wg signalling is essential for the development of the wing blade [3], and one of its key target genes in the third larval instar disc is *senseless* (*sens*), whose expression flanks the Wg signalling source along the presumptive wing margin [36] (figure 5*a*). If Axin–GFP is expressed in the presumptive wing blade, abundant large cytoplasmic puncta are observed in the epithelium (probably degradasomes, since each punctum also contains E-APC, electronic supplementary material, figure S2*a*, arrowheads). Evidently, these degradasomes are highly active, as the presumptive wing blade territory is substantially reduced upon overexpression of Axin–GFP in wing discs, which causes cell-autonomous repression of *sens* to almost undetectable levels (figure 5*b*). In contrast, *apc* null mutant wing disc clones show strong and cell-autonomous derepression of *sens*, even in the presence of Axin–GFP (figure 5*c*; electronic supplementary material, figure S2*b*), whose function in repressing this Wg target gene is evidently abrogated upon APC loss-of-function, paralleling our results in *apc* null mutant embryos. However, the level of *sens* expressed is reduced in *apc* null mutant cells that co-express Axin–GFP with *dsh*RNAi (figure 5*d*, left; electronic supplementary material, figure S2*c*). Indeed, the Sens staining in the *apc* mutant territories of these discs inversely correlates with GFP fluorescence (marking co-expression of Axin–GFP and *dsh*RNAi), indicating that the depletion of Dsh restores some of the Axin–GFP function in repressing this Wg target gene.

Examining the subcellular distribution of Axin–GFP in these disc epithelia, we noticed a dramatic apical re-distribution of the bulk GFP fluorescence: in every single *apc* null mutant clone (*n* = 57, from 10 wing discs), we observed numerous small Axin–GFP puncta associated with the apical PM (figure 5*e*, arrows; electronic supplementary material, figure S2*d*,*e*). Adjacent wt territories contain far fewer PM-associated puncta, which are also smaller than the cytoplasmic degradasomes in these wt cells (figure 5*e*, arrowheads). Importantly, there are barely any PM-associated puncta in *dsh*; *apc* double-null mutant clones, in which we predominantly observe diffuse cytoplasmic Axin–GFP (figure 5*e*, asterisk). Therefore, most (if not all) Axin–GFP puncta that form in the absence of APC are re-located to the apical PM by Dsh, as in the embryo (figure 3*b,c*; see also electronic supplementary material, figure S1*a*). This Dsh-dependent PM relocation could help in explaining why Axin–GFP is inactive in *apc* null mutant cells (figure 5*c*), and why its function is restored by depletion of Dsh (figure 5*d*). Our results indicate that APC enables Axin to function, at least in part, by shielding it from Dsh- dependent recruitment to the PM, into signalosome-like particles, which normally block its function (see §2).

### 3.5. Adenomatous polyposis coli displaces Dvl2 from cytoplasmic Axin protein assemblies

**Figure 6.**
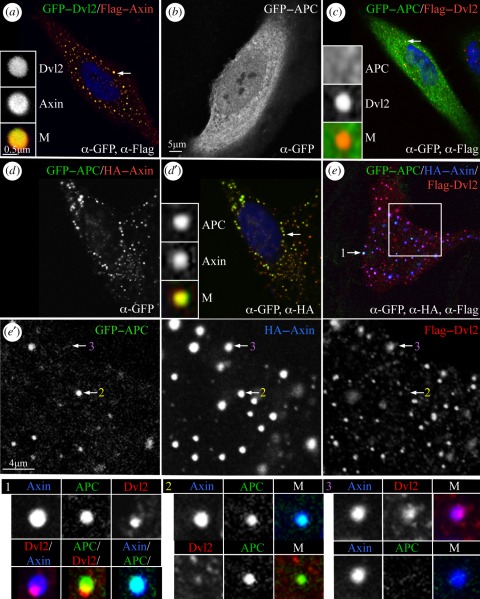
APC displaces Dvl2 from Axin assemblies. Single confocal sections through transfected HeLa cells, after fixation and antibody staining, expressing tagged proteins as indicated in panels (*a,c,d*',*e*), merges (*b,d,e*') single channels (stained with antibodies against tags; *e*', boxed area of *e*, at higher magnification; single channels are shown); insets on the left show co-localizations within individual puncta (indicated by arrows; M, merges). Shown at the bottom are the two types of double-positive puncta, Axin–APC and Axin–Dvl2 (examples 2 and 3 in *e*'), and triple-positive puncta (example 1 in *e*) invariably exhibiting epicentric Dvl2 clusters (see also electronic supplementary material, figure S3).

Our results described above indicate that APC opposes constitutively the recruitment of Axin by Dsh, implying that it can compete with Dsh for its interaction with Axin. This is somewhat surprising, given that Axin binds to APC through its N-terminal RGS domain and to Dvl2 through its C-terminal DAX domain, so should be able to interact simultaneously with both binding partners. To examine the mutual interactions between these three proteins, we co-expressed them in HeLa cells to monitor their co-localization in puncta. As expected [13,37], Axin and Dvl2 by themselves each form DIX-dependent puncta, which co-localize precisely and concentrically upon co-expression (figure 6*a*). APC on its own is distributed diffusely throughout these cells (figure 6*b*), which remains unchanged upon co-expression with Dvl2 (figure 6*c*), confirming that APC and Dvl2 do not interact. However, as previously demonstrated [10], APC is recruited efficiently into Axin puncta upon co-expression (figure 6*d*).

Strikingly, if all three proteins are co-expressed (figure 6*e*), we never observe puncta with comparable and concentric signals of all three proteins. Indeed, the only puncta with substantial levels of all three proteins exhibit concentric APC–Axin signals, but peripheral Dvl2 clusters (figure 6*e*, example 1; *n* = 44, four cells scored). In addition, we observed concentric Axin–APC puncta (figure 6*e*', example 2; *n* = 30) as well as concentric Axin–Dvl2 puncta (figure 6*e*', example 3; *n* = 73), and also many puncta that contain exclusively Dvl2 (*n* = 58). This indicates that Axin cannot interact simultaneously with APC and Dvl2, and that APC competes with Dvl2 for its interaction with Axin under these conditions. Indeed, the epicentric Dvl2 in the triple-positive puncta suggest that these puncta are ‘transitional’ assemblies in which Dvl2 is being displaced from Axin by APC. Note that we have not tested whether the converse would also be true, namely whether Dvl2 would displace APC from Axin in membrane-associated signalosomes, as our co-expression experiments were done in the absence of Wnt stimulation.

Similar results were obtained with HC, a minimal Axin-binding fragment from the central third of APC that is fully active in complementation assays of *APC* mutant colorectal cancer cells [38]. As with full-length APC, the only triple-positive puncta we observed (*n* = 96; three cells scored) exhibit concentric Axin–HC signals but epicentric Dvl2 (electronic supplementary material, figure S3, arrow). In addition, we see numerous Dvl2-only puncta, and also many Axin–HC puncta, but Axin–Dvl2 puncta are rare. Thus, the minimal Axin-binding APC fragment appears to be highly active in displacing Dvl2 from Axin—indeed, more active than full-length APC, perhaps because of its high expression levels [38].

## 4. Discussion

We used *Drosophila apc* null mutants [30] for stringent *in vivo* function tests to show that APC is indispensable in *Drosophila* tissues for Axin's activity in assembling functional degradasomes that destabilize Armadillo. Our evidence suggests that the same is also true for *APC* mutant colorectal cancer cells in which Axin–GFP, if expressed at low levels, shows a marked tendency to fail in assembling functional degradasomes. This provides a new insight into how APC promotes the destabilization of Armadillo/β-catenin—namely by enabling Axin to assemble degradasomes. The failure of this assembly step explains why Axin fails to destabilize Armadillo/β-catenin, given that this function of Axin crucially depends on its DAX-dependent polymerization [11]. We emphasize that previous studies (including our own) have shown that APC loss-of-function can be bypassed by Axin overexpression [10,18–20,22], which indicated a non-obligatory role of APC in the destabilization of β-catenin/Armadillo. However, these studies underestimated APC's essential role in this process since Axin was assayed under conditions of residual APC function, and possibly also since high Axin overexpression levels were used for complementation.

Why does Axin fail to assemble functional degradasomes in the absence of APC? We believe that this is due to a combination of two different effects of APC loss on Axin. First, Axin is destabilized in the absence of APC (figure 1*l*), so its levels may fall below the minimal cellular concentration required for DAX-dependent polymerization: note that the DIX domain auto-affinity is in the micromolar range [14], and so the DAX-dependent polymerization may not occur spontaneously at low cellular Axin concentrations, but might require a co-factor capable of clustering Axin, increasing its local concentration and nucleating polymerization [11]. APC is a candidate for such a co-factor, given its relatively high cellular abundance and affinity to Axin [23,24,32,39] which allow it to associate efficiently with Axin at physiological concentrations. APC might cluster Axin directly, by binding simultaneously to multiple Axin molecules through its multiple Axin-binding sites [23], or indirectly through additional factors (such as CtBP, itself capable of clustering APC [40]). Notably, Axin would become independent of this co-factor if overexpressed at high enough levels, as this would allow it to overcome its low auto-affinity and to polymerize spontaneously.

Second, the absence of APC (or of binding to APC) renders Axin prone to Dsh-dependent relocation from the cytoplasm to the PM (figures 3*b*,*c* and 5*e*), into signalosome-like particles. Since the recruitment of Axin into signalosomes normally blocks its function in promoting the phosphorylation and destabilization of β-catenin/Armadillo (see §2), its relocation to the PM might also explain its inactivity in the absence of APC. Our evidence indicates that APC shields Axin from interaction with Dsh in the absence of Wnt signalling, to ensure Axin's function in the cytoplasmic degradasomes. This shielding function of APC may be particularly important in cells experiencing non-canonical Wnt signalling (promoting PM-association of Dishevelled), such as in third-larval instar wing discs [41] (figure 5), and possibly in late-stage embryos [42] (electronic supplementary material, figure S1*a*). Our observations in *Drosophila* tissues suggested that APC may compete with Dishevelled for association with Axin, which is strongly supported by our evidence from co-expression experiments in mammalian cells (carried out in the absence of Wnt stimulation): these indicate that Axin cannot interact simultaneously with APC and Dvl2, and that APC is capable of displacing Dvl2 from Axin protein assemblies (figure 6; electronic supplementary material, figure S2). The notion of a competition between APC and Dishevelled for their association with Axin is consistent with previous evidence from epistasis experiments in *Drosophila* embryos [31] and murine intestinal tumours [43], which indicated that APC acts at the same level as Dishevelled rather than below it.

Evidently, the function of APC that shields Axin from its interaction with Dishevelled is somehow antagonized by Wnt stimulation, which enables Dishevelled to bind to Axin and recruit it to the PM into signalosomes [17,18]. Indeed, Wnt signalling may overcome the competition between APC and Dishevelled for their binding to Axin, allowing simultaneous interaction of all three proteins. Consistent with this, Axin–GFP appears to co-localize with E-APC in the Wg signalling zones of *Drosophila* embryos within the PM-associated signalosomes (figures 1*j* and 3*a*) that are likely to also contain Dsh (although we have not been able to confirm this directly owing to the lack of a suitable Dsh antibody), suggesting that Wg signalling allows all three proteins to coincide in signalosomes.

We note that a previous study in *Drosophila* uncovered a positive role of APC in *antagonizing* Axin (rather than promoting its function), thereby stimulating signalling through Armadillo [44]. The key evidence supporting this rather unexpected conclusion was that the levels of Axin were upregulated in *apc* null mutant wing disc clones, as shown by immunofluorescence [44]. This contrasts with our own result from *apc* mutant embryos, which show much reduced levels of Axin–GFP, as judged by Western blotting (figure 1*l*). Although this quantitative biochemical approach is difficult to apply to *apc* mutant wing disc clones (owing to insufficient *apc* mutant material), the dramatic PM relocation of Axin–GFP we observed in these clones (figure 5*e*; electronic supplementary material, figure S2*d*,*e*) suggests that Takacs *et al*. [44] may have been misled by the high levels of apical Axin, and mistaken these for a general upregulation rather than simply a relocation (as we have shown for Axin–GFP). Our own evidence reaffirms the negative role of APC in Wg/Armadillo signalling, demonstrating an essential function of APC in keeping Axin in the cytoplasm, where it enables it to assemble functional degradasomes.

The main corollary of our results from *Drosophila* tissues is that APC promotes the DAX-dependent homopolymerization of Axin (required for degradasome assembly), and that it antagonizes the heteropolymerization between Axin and Dishevelled (mediated by DIX–DAX interaction) [11]. The latter is further supported by our evidence from co-expression experiments in mammalian cells that APC displaces Dvl2 from Axin puncta (figure 6; electronic supplementary material, figure S3). This creates a mechanistic conundrum: APC binds to the N-terminal RGS domain of Axin, but appears to control the DAX-dependent interactions at its C-terminus. Although it is conceivable that APC achieves this at ‘long range’, given its unusually large size we think it more likely that APC relies on additional factors, perhaps even on enzymes, to promote Axin's self-assembly at the expense of its heteropolymerization with Dishevelled. Future work will be required to determine the precise molecular mechanism by which APC enables Axin to assemble functional degradasomes and opposes its recruitment by Dishevelled, and how this is overcome during Wnt signalling.

## 5. Material and methods

### 5.1. Plasmids

The following plasmids were used: human Axin–GFP, HA–Axin, Flag–Axin, Flag–Dvl2, GFP–Dvl2 [13,37]; human GFP–APC, GFP–HC [38,39]; *Drosophila* Axin–GFP [18]; mutants were generated by standard QuikChange mutagenesis, and checked by sequencing.

### 5.2. *Drosophila* strains and analysis

The following mutants and transgenic strains were used: *UAS.Axin-GFP*, *arm.GAL4*, *dsh*^*v*^^26^ [18]. Mutant germline clones were generated by standard procedures, and Axin-expressing *apc* null mutant embryos were selected on the basis of GFP, but lacking RFP, from the progeny of the following cross: *hs-flp*/+; *arm.GAL4*/+; *FRT82B dApc*^*Q*^^8^ *e-apc*^*f*^^90^/*FRT82B ovo*^*D*^ × *UAS.Axin-GFP/Cyo*; *FRT82B dApc*^*Q*^^8^ *e-apc*^*f*^^90^/*tub-RFP*. Rescue of *axin* null mutant embryos [7] was done as described by Fiedler *et al*. [11]. Double-mutant *dsh*^*v*26^; *dApc*^*Q*^^8^ *e-apc*^*f*^^90^ wing disc clones were generated with *vg.GAL4 UAS.flp* [45], by dissecting discs from female *TM6*^+^ larvae from the cross *FRT101 dsh*^*v*^^26^/+; *UAS.Axin-GFP/Cyo-TM6*; *FRT82B dApc*^*Q*^^8^ *e-apc*^*f*^^90^/*Cyo-TM6* × *lacZ FRT101*/Y; *vg.GAL4 UAS.flp*/*Cyo-TM6*; *FRT82B lacZ*/*Cyo-TM6* (the compound chromosome *Cyo-TM6* ensures that all discs with *apc* mutant clones also express Axin–GFP); double mutant clones were identified by lack of nuclear β-galactosidase staining, *apc* single mutant clones (that are *dsh*^+^) by lack of cytoplasmic puncta (several per cell) plus appearance of prominent apical GFP puncta. Dsh depletion was done by co-expressing *dsh*RNAi [46] with Axin–GFP (by *vg.GAL4 UAS.flp*, to generate *apc* null mutant clones, as described above); Dsh depletion under these conditions is probably partial since the expression of *dsh*RNAi by itself (with *vg.GAL4 UAS.flp*) neither affects the development nor the patterning of the wings. Antibody staining of paraformaldehyde-fixed embryos and wing discs was done as described previously [18,47], with the following antibodies: α-Armadillo [48]; α-E-APC [32]; α-β-galactosidase (Promega); α-Sens [49]; α-Wg (Developmental Studies Hybridoma Bank). Confocal microscopy was carried out with an Axio imager upright microscope (Zeiss 510 confocal scanhead) using single confocal scans and standardized settings throughout. Embryonic cuticles and wings were prepared and viewed by standard procedures. For Western blots, GFP-positive 3–16-h-old embryos were hand-picked under a dissecting microscope, and embryonic extracts were prepared and processed as described by Hamada & Bienz [29]; two independent experiments were done.

### 5.3. Cell-based assays

SW480 and SW480 + APC cells [34] were kindly provided by Maree Faux. These cells, and HeLa cells, were grown in Dulbecco's Modified Eagle Medium supplemented with 10 per cent foetal bovine serum, and transfected in 6-well plates with Lipofectamine 2000 (Invitrogen). To keep Axin expression levels low, 100 ng per well of Axin-expression vectors were used (supplemented with pRL-TK plasmid to 500 ng per well). Cells were fixed 18 h after transfection, and stained with α–β-catenin (rabbit antiserum), α-GFP, α-HA or α-Flag antibodies (Sigma), as described previously [39]. High Axin–GFP expression levels (figure 2*a,e*) were detectable at a low laser setting (i.e. 5% of the 488 nM laser), whereas the reliable detection of low Axin–GFP expression levels above background (figure 2*b*–*d*,*f*) required a higher setting (i.e. 30% of the 488 nM laser).
